# Implications of sperm heat shock protein 70-2 in bull fertility

**DOI:** 10.14202/vetworld.2022.1456-1466

**Published:** 2022-06-13

**Authors:** Zulfi Nur Amrina Rosyada, Mokhamad Fakhrul Ulum, Ligaya I. T. A. Tumbelaka, Dedy Duryadi Solihin, Bambang Purwantara, Erdogan Memili

**Affiliations:** 1 Reproductive Biology Study Program, Postgraduate School, IPB University, 16680, Bogor, Indonesia; 2 Department of Veterinary Clinic, Reproduction and Pathology, School of Veterinary Medicine and Biomedical Sciences, IPB University, 16680, Bogor, Indonesia; 3 Department of Biology, Faculty of Science, IPB University, 16680, Bogor, Indonesia; 4 Agricultural Research Center, College of Agriculture and Human Sciences Prairie View A&M University, Prairie View, TX, USA

**Keywords:** fertility, heat shock protein, protective, semen quality, stress

## Abstract

Heat shock protein 70 (HSP70) is one of the most abundant chaperone proteins. Their function is well documented in facilitating the protein synthesis, translocation, de novo folding, and ordering of multiprotein complexes. HSP70 in bovine consists of four genes: HSP70-1, HSP70-2, HSP70-3, and HSP70-4. HSP70-2 was found to be involved in fertility. Current knowledge implicates HSP70-2 in sperm quality, sperm capacitation, sperm–egg recognition, and fertilization essential for bull reproduction. HSP70-2 is also involved in the biological processes of spermatogenesis, as it protects cells from the effects of apoptosis and oxidative stress. Fertilization success is not only determined by the amount of sperm found in the female reproductive tract but also by the functional ability of the sperm. However, subfertility is more likely to be associated with changes in sperm molecular dynamics not detectable using conventional methods. As such, molecular analyses and omics methods have been developed to monitor crucial aspects of sperm molecular morphology that are important for sperm functions, which are the objectives of this review.

## Introduction

Bull fertility is a significant factor in determining the total pregnancy rate [1]. The superior bull required for artificial insemination (AI) can produce ~100,000 straws of semen with the potential to carry out AI and can fertilize oocytes in thousands of cows each year [2]. Thus, the quality of frozen semen has an essential role in the AI process. Therefore, examination or assessment of fertility capacity needs to be evaluated in ejaculated semen for acrosome integrity and sperm mass motility. The progression of sperm motility and mobility and the post-thaw survivability of sperm are considered before commencing a breeding program or AI. In contrast, visual examination of semen samples may not be adequate to predict bull fertility accurately [3]. Several recent studies [4, 5] have shown the distinction between the functional properties of sperm and sperm compounds, such as mRNA and small non-coding RNA, proteins, and sperm metabolites, between sperm from bulls with varying fertility levels.

Chen *et al*. [6] showed that mRNA for heat shock protein (HSP) 70 (HSP70) is involved primarily in biological activities, such as energy metabolism processes for sperm motility and protein processing in sperm populations. Motiei *et al*. [7] showed that HSP70 is critical in spermatogenesis, specifically in sperm meiosis and maturation and sperm–egg recognition. Gilbert *et al*. [8] reported that degraded and undamaged transcripts coexist in sperm mRNA population translated into functional proteins, namely, HSPs and other functional and non-functional proteins. Ikwebuge *et al*. [9] reported that HSPs protect cells from apoptosis damage and oxidative stress.

Numerous genes regulate sperm motility, of which expressed products are associated with the structure of the flagellar membrane, the cell’s energy metabolism, operation of the mitochondria, and ion exchange channels. With remarkable advancements in transcriptomic and proteomic analyses, a faster and more reliable technology can be developed to determine the sperm’s reproductive potential [10]. This review summarized the identification of HSP markers for bull fertility rates and their correlation with sperm quality. This scientific resource also outlined the critical role of *HSP70*-2 in sperm quality and its subsequent functional transformation during capacitation. The knowledge highlights the potential of HSP70-2 as a clinically useful marker of sperm quality and correlates with sperm maturity, function, and fertility. This review emphasizes the need for further analyses of this chaperone protein, providing essential insights into the molecular mechanisms that regulate sperm physiology.

## Background on HSPs as Molecular Chaperones Proteins

Chaperone proteins are a complex family of proteins with various structural characteristics [11, 12]. Because chaperone proteins provide cellular tolerance to environmental stressors, the cell stress response, more often known as HSPs, refers to the majority of the chaperone protein family’s members [13]. HSPs are the most abundant chaperone family member. They are involved in protein synthesis, translocation, and *de novo* folding. Assembling multiprotein complexes is another critical role [14].

The ability of chaperone proteins to create oligomeric complexes is crucial, as breakthroughs in functional proteomics have enabled the demonstration that most cell proteins work better as multiprotein complexes than as single proteins [15]. The relevance of specific chaperone protein functions spans from classic protective roles to controlling regular cellular operations, including metabolism, growth, differentiation, and apoptosis; chaperone protein functions characteristic is highlighted by the overlapping expressions of HSPs [16, 17]. The capacity of molecular chaperone proteins to recognize and interact with the client protein’s exposed hydrophobic regions allows them to perform these diverse functions. These interactions prevent erroneous protein association or aggregation and guide proteins through folding, transport, or degradation pathways [18].

High-temperature stress, chemical or physical stress, viral infections, medicines, and modifying agents cause HSP production in cells. Modifying agents that cause HSP production in cells can appear through hyperthermia, oxidative stress, metabolic challenge, and aging. The cytoprotective functions of these proteins include protein homeostasis and apoptosis inhibition [19, 20]. Stress-induced HSP aids in the recoating of denatured proteins, preventing the accumulation of harmful metabolic effects of misguided (misfolded) protein and cell death caused by proteases [21]. Physiological activities, such as proliferation, differentiation, development, and aging, might stimulate HSP production [22].

HSP is classified into six families in mammals: HSP100, HSP90, HSP70, HSP60, HSP40, and HSP27 [23]. The protected chaperone protein family has at least 14 members in the human genome, including HSP70 [24]. The HSP70, also termed as HSPA family, consists of at least 14 members: HSPA1A, HSPA2/HSP70-2, HSPA4, HSPA5/BiP HSPA6/HSP70B, HSPA7/HSP70B, HSPA8, HSPA9, HSPA12A, HSPA12B, HSPA13, HSPA14, HSPA1LB, and HSPA1L [25]. HSP70 in bovine also includes four genes: *HSP70-1*, *HSP70-2*, *HSP70-3*, and *HSP70-4*. The HSP family Chaperone proteins have been found in the sperm of various animals, including mice [26], humans [27], pigs [28, 29], cattle (bovine) [30], and goats [31]. The most abundant proteins of the HSP family, especially HSP60 member 1 were also found in ram sperm [32].

Cochaperone HSP100 with HSP40, HSP70, and HSP90, functions in the refolding of aggregates. Nuclear Chaperone proteins alleviate protein aggregation in the nucleus. HSP100 is a nuclear-localized chaperone and interacts with HSP70 to solubilize and rescue aggregated proteins. In contrast, HSP110 Chaperones are homologous to HSP70, but they are larger and lack chaperone activity. HSP110 chaperones also localize to nuclear protein aggregates and partner with HSP70 to function as disaggregates. Thus, HSP100 and HSP110 may be important for protecting against diseases that involve nuclear protein aggregates [33, 34]. HSP110 also works with HSP70 to help store proteins and protect cells from damage [27]. The alternative classification uses the cochaperone HSP100 function to categorize HSPs: (a) Chaperone proteins (HSP70 and HSP60), (b) proteins with catalytic activity (ubiquitin, HSP100, protease, and tyrosine phosphatase), and (c) proteins with unclear functions (α-crystals and secreted glycoproteins) [35]. Large molecular weight of HSPs (HSP60, HSP70, HSP90, and HSP100) are adenosine triphosphate (ATP) dependent and act in various ways, including protein folding and translocation, cytoprotection, core hormone receptor control, and apoptosis regulation. In contrast, smaller HSPs (HSP10 and HSP40) are ATP independent and tissue-specific, play a critical role in folding proteins and have antiapoptotic properties [24].

The primary role of HSP70 is to bind to denatured or partly synthesized peptide sequences, preventing aggregation and enabling the chaperone to refold. HSP70 facilitates transmembrane trafficking by stabilizing client proteins in a semi folded form and forming functional complexes from these proteins [36]. Besides their protective function against stress, HSP70 is one of the main parameters affecting the sperm’s ability to fertilize [28]. One study evaluated *HSP70-2* expression in ejaculated sperm from 10 men with oligozoospermia revealed lower expression levels in infertile sperm [37]. Along with Zhang *et al*. [38], these observations demonstrated that motility is crucial in determining the sperm’s ability to fertilize. *HSP70*-2 expression levels can affect sperm motility. *HSP70*-2 also plays a critical regulatory role in the numerous stages of sperm development and maturation and modulation of fertilization [39].

Recent research on postmeiotic germ cells showed that *HSP70*-2 takes on an unexpected role as a chaperone for the DNA-packing transition protein unique to spermatids [40]. *HSP70*-2 also functions as an intermediate; protamines are replaced with histones during spermiogenesis. These results indicated that *HSP70*-2 is required to properly regulate chromatin remodeling in differentiating spermatids. In the absence of HSP70-2, aberrant sperm with weakly compacted chromatin is formed, corresponding to H4 histone overexpression [41]. The coordinated action of chaperone molecules contributes to the activation of receptor proteins on the exterior of sperm to initiate sperm–egg recognition events [26, 40].

## HSP70 Localization in Sperm

In mammalian testicles and sperm, several members of the HSP70 gene family have been confirmed to be present. In mouse germ cells, HSP70-2 is required for meiosis [42, 43]. HSPs have been discovered on the sperm membrane’s surface [44]. Immunofluorescence analysis demonstrated that the HSP70 antigen is ubiquitous on the surface of the human sperm [45]. During capacitation and acrosome reaction in bull sperm, HSP70 was identified in the acrosome and postacrosome areas [46]. In one experiment, trypsin was used to trypsinize motile sperm on their soft surfaces, and peptides were liberated and extracted from the medium using high-performance liquid chromatography. Furthermore, tandem mass spectrometry analysis confirmed four *HSP70*-2 peptides and four HSPC1 peptides among the enzymatically generated products, supporting the hypothesis that these Chaperone proteins were positioned on the surface of the sperm membrane. Thus, HSP70 was found in mature male sperm; protein redistribution occurs during capacitation and acrosome reactions. HSP70 in the acrosome may help stabilize sperm plasma membrane proteins. Another immunofluorescence study demonstrated that *HSP70*-2 is localized to the plasma membrane in the cauda epididymis [45, 46]. In recent proteomic research, human sperm membrane fraction was determined using two-dimensional gel electrophoresis and human plasma anti-sperm antibodies were used to identify the proteome. Mass spectrometry was applied to identify two membrane autoantigens, HSPA2 and HSPA1L [47, 48, 49].

Sperm can acquire HSP70 through epididymal secretion. The epididymal maturation of sperm proteins may be required to develop the fertilized potential. Fertilization occurs when a spermatozoon binds to and fuses with an oocyte’s plasma membrane in the oviduct [48, 50]. Carreira and Santos [13] demonstrated HSP70 in bovine spermatogonia, spermatids, and sperm. The introduction of this molecule modifies HSP70 intracellular position during spermatogenesis, after ejaculation, and during capacitation.

## Functional Mechanisms of HSP70 Actions

### HSP in apoptosis

**Figure-1:**
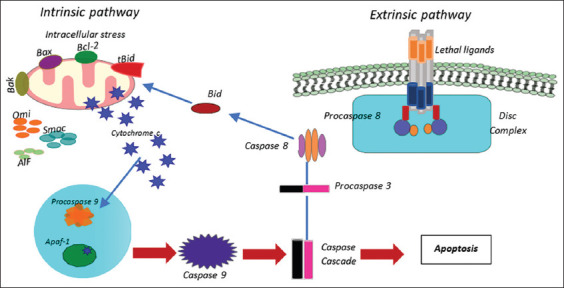
Pathways of apoptosis. [52].

An evolutionary cause for cell death is characterized by a distinct series of occurrences involving biochemistry and morphology that end in an ordered cell elimination [51] through caspase-dependent apoptosis (Figure-1). Favaloro *et al*. [52] demonstrated that blebbing phenomena arise because of a cascade of molecular signaling. Kourtzelis *et al*. [53] stated that phagocytic cells efficiently detect resultant apoptotic cells without generating inflammation or damage. Cell death mediated by caspases is conditional on caspase activation, which cleaves several substrates [54] due to biochemical and morphological modifications, such as apoptotic and nonapoptotic, which are typical of this type of death.

Because caspase activation occurs through two distinct molecular routes, apoptosis is both an extrinsic and intrinsic process. The evolutionary sequence of molecular signaling processes has evolved in multicellular creatures to respond to cellular damage and environmental stimuli. Chaperone proteins are members of the HSP subfamily, such as Hsp60, Hsp70, and Hsp90, which are beneficial for preventing apoptosis. In addition to folding and assembling proteins, Chaperone proteins are known to transport proteins between different locations in the body. As a result, apoptosis is regarded as usual and necessary because of various physiological mechanisms (cell death, cell division, and cell injury). The stress response due to heat shock modulates the balance between dead cells and cells capable of sustaining survival [55].

By interacting with keystone proteins at three different sites, HSPs can inhibit both intrinsic and extrinsic apoptosis: (i) Upstream of mitochondria, regulating nerve signaling; (ii) regulating apoptogenic particle release in mitochondria; and (iii) postmitochondrial survival-enhancing medicines or proteins inhibiting apoptosis. Thus, HSP is a protein that prevents apoptosis that is crucial for cell survival [56].

Some stimuli trigger HSP, both internally (intrinsic) and externally (extrinsic) [57]. HSP blocks caspase activity that prevents apoptosis. Hsp27, Hsp70, Hsp60, and Hsp90 overexpression has been shown to suppress apoptosis and prevent caspase activation in a range of cellular models after exposure to a wide range of cellular stress, such as misfolding protein accumulation and DNA damage that is caused by reactive oxygen species (ROS) [58].

**Figure-2:**
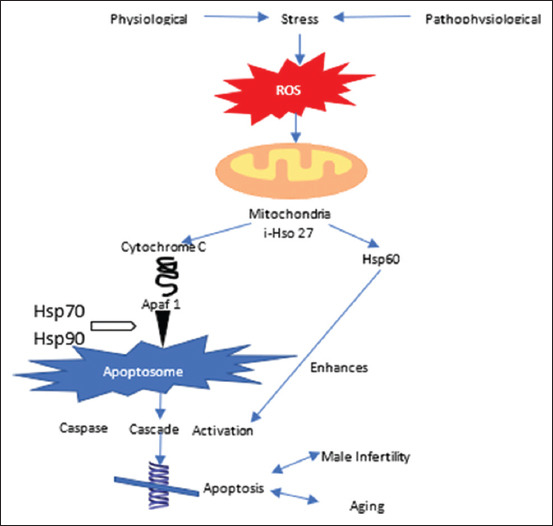
Involvement of heat shock proteins in human aging and fertility. [19].

There are two types of functional cases: Caspases up and down. Upper-flow caspase activation occurs when many enzyme molecules appear close together and bind to activation-coordination chemicals, causing full cleavage and activation [59]. Figure-2 highlights the involvement of HSPs in male aging and fertility. Purandhar *et al*. [19] demonstrated that the cleavage of the upstream caspase prodomain affects HSP infertility, aging, and apoptosis in males.

The spermatogenesis process involves HSP/HSP4 proteins [43] and the ubiquitin/proteasome pathway [39], regulating the folding and sorting of proteins during spermatogenesis. The complexity of spermatogenesis must be due to cell differentiation and the processes of mitosis, meiosis, and cell division. Through endocrine mechanisms (primarily regulated by hypothalamic, pituitary, and gonad steroids) [60], there are intragonad regulatory networks that allow communication among the cellular, intracellular, and local environments surrounding a cell [61].

Two HSP70 members are expressed in spermatogenic cells; specifically, the *HSP70-2* gene (now referred to as HSP70-A2) is expressed during meiosis [62], whereas HSP70t (HSPA1L) is described postmeiotically [63]. *HSP70-2* gene mutations cause infertility, as manifested by the widespread death of pachytene spermatocytes and sperm loss [43]. *HSP70*-2 is associated with complex synaptonemal tasks; indeed, *HSP70*-2 deficiency results in chromosomal pair split (desynapse) [64]. Apoptosis in germ cells has also been described as a result of *HSP70*-2 deficiency in testicular injury caused by oxidative stress [65,66]. Transition proteins 1 and 2 are important packaging proteins for spermatid DNA [67]. The acrosomal surface of sperm contains *HSP70*-2, and *HSP70-2* is not expressed in patients with idiopathic insufficiency, resulting in a failure of sperm–egg recognition [68].

HSP60, HSP70, and HSP90 immunolocalization in sperm are associated with sperm-fertilizing ability in pigs, horses, dogs, and cats [69]. HSP70 and HSP90 were found in identical locations in rabbits in spermatid Stages VII and VIII and the spermatogonia plasm, whereas *HSP70*-2 was found in the pachytene cytoplasm [70]. Heat shock transcription factor deficiency is associated with reproductive issues and regulates the dynamic HSP expression during spermatogenesis, including the etiology of human idiopathic azoospermia [71].

### Functional mechanisms of *HSP70* in chaperone protein folding

Heat and other stressors are thought to cause considerable chaperone expression. It is proposed that the sperm surface membrane contains members of the families of HSP60, HSP70, and HSP90. The chaperone protein families of HSP60, HSP70, and HSP90 play important roles in spermatogenesis [72].

*HSP70*-2 is produced in synaptonemal spermatocytes and can be resynthesized throughout the last stages of spermiogenesis, coupled with cytoplasm expulsion and reformation of the sperm plasma membrane which involves the translocation of sperm protein [73]. *HSP70*-2 works with other Chaperone proteins expressed in the testes [74]. *HSP70*-2 is important in spermatogenic differentiation [75]. Apoptosis lowered chaperone expression (*HSP70*-2) and higher in DNA fragmentation [76], a deficiency in the substitution of histones for protamines [77], higher rates of oxidation lipids [73], and an increase in chromosomal aneuploidy [75].

HSP can prevent the accumulation of rearranged proteins by various types of stressors [21]. Decreased HSP70 expression discovered by Zhang *et al*. [78] retained the protein structure throughout the freezing–thawing process. Zhang *et al*. [38] also demonstrated that the integrity of the membrane was decreased after freezing–thawing processes; this may be attributed to a decrease in HSP70 expression. HSP70 interacts with lipids and may be involved in folding and transporting membrane proteins [79].

The decrease in HSP70 protein synthesis could be caused by reduced HSP70 expression levels. As a result of the decreased activity of HSP70, aberrant protein folding occurs in the sperm membrane. Sperm membrane fluidity may be impaired in several cases. Aboagla and Terada [80] showed that the fluidity of the sperm membrane is regulated by abundant membrane proteins. As stated previously, decreased sperm motility is assumed to be caused by a reduction in the fluidity of the sperm membrane.

### Effect of freezing–thawing on the functioning mechanism of HSP70

Zhang *et al*. [78] discovered that freezing–thawing reduces sperm motility, membrane integrity, and HSP70 expression levels (p < 0.05). These data implied that low HSP70 expression levels might cause decreased sperm motility. Further examination of a decrease in HSP70 expression during freezing–thawing of bull sperm is required.

HSP70 family members (HSP70) are molecular bodyguards capable of regulating folding, transporting, and assembling proteins in complexes [9] under stressful heat and normal physiological conditions. The pro-folding activity of the HSP70 family is mediated by the ATP-binding cycle, hydrolysis, and exchange [81]. HSP70 activity is dependent on the recruitment of cochaperone proteins from the conserved HSP40/DNAJ protein family on a molecular level [82].

The HSP family performs the role of a molecular chaperone, preventing protein aggregation and modulation of protein stability and shape. HSPs, including the HSP40/DNAJ protein, are stored in prokaryotes and eukaryotes [80, 81]. Cellular damage or diseases, such as neurodegenerative disorders and infertility, result from protein misfolding caused by cell damage [82].

During freezing–thawing, damage to the sperm membrane reduces the sperm’s quality. Because of the physical and chemical stress caused by the freezing–thawing process, it is believed that these processes may induce sperm plasma membrane damage due to oxidative stress [83, 84]. By controlling the activity of many enzymes in sperm cells, HSP70 can govern cell function. Zhang *et al*. [38] reported that when sperm motility decreased, HSP70 expression levels also decreased. HSP70-2 located in the plasma membrane to the midpiece of the sperm (found in the mitochondria), functions as a cell protector and energy regulator for cell motility. When the level of HSP70-2 expression is altered, it may reduce the function of antioxidant enzymes in cells, allowing substantially more ROS to harm sperm and reduce motility.

Stress activates protein families that have previously been triggered by homologous stress, such as kinases, and HSP70 is sufficient to suppress the function of these protein kinases. Thus, HSP70 enables cells to fight apoptosis without homologous stress [85]. Elevated HSP70 expression levels in cells resist protein kinase activity that reinforces resistance cells. It was concluded that sperm motility is positively correlated with HSP70 expression levels (ranging from 0.327 to 0.785; Chen and Xu 1998 in Zhang *et al*. [78]). Meanwhile, Zhang *et al*. [78] and previous studies suggested that increased HSP70 production may help sperm cells survive cryopreservation. Freeman *et al*. [86] stated that HSP70 could boost Ca^2+^-ATPase activity, minimize membrane damage, and reduce the effects of stress-induced reactions on mitochondria.

When cells are heated, HSP70 increases superoxide dismutase (SOD) activity, shielding them from oxidative damage. HSP70 regulates SOD activity to protect sperm membranes from ROS. Stress activates eukaryotic initiation factor 2 (eIF-2), which regulates protein production. Stress causes eIF-2 to be dephosphorylated, allowing other proteins to be synthesized and sperm cells to operate normally. Rio *et al*. [46] demonstrated that the amount of HSP70 expression was reduced because of freezing–thawing processes. The decrease in HSP70 levels may explain why the sperm cells’ function is compromised beyond their resistance. HSPs have been demonstrated to include an intrinsic ATPase required for the *in vivo* activation of native client proteins. Mayer and Bukau [25] showed that the hydrolysis of ATP is significant for the protein chaperone HSP70. The sperm motility and complex mitochondrial respiratory activity are positively correlated [87]. When sperm lack energy to survive, their motility can diminish.

Furthermore, ATPase activity is temperature-dependent, and cold shock can reduce ATPase activity. Thus, reduced ATPase activity reduces sperm motility. A decrease in temperature is known to cause a reduction in sperm motility and HSP70 expression levels. As shown by the positive link between HSP70 expression levels and the three stages of sperm motility (ranging from 0.328 to 0.785), highly motile sperm had higher levels of HSP70 expression. Sperm has sufficient energy to produce increased amounts of HSP70 and sustain motility after freezing–thawing, due to increased HSP70 expression, which is resistant to ATP breakdown [78].

## Roles of HSP in Fertilization

This HSP Chaperone plays a critical role in spermatogenesis, reconstituting sperm morphology via cytoplasm extrusion. The sperm plasma membrane must be remodeled to penetrate oocyte cumulus cells and contact the zona pellucida (ZP). There is a shortage of knowledge regarding the mechanisms underlying the development of ZP binding sites. The amount of zone binding sites and hyaluronic acid receptors in immature human sperm is limited, resulting in a reduction in fertilization potential due to the lower chaperone production generated by the testes, namely, 70-kDa *HSP70*-2 [73].

### Molecular Chaperone proteins in gamete fusion

The fusion of sperm and oocytes occurs when the inner acrosomal membrane of the sperm cell and the microvilli-rich areas of the oocyte plasma membrane come together [88]. It is hypothesized that, given the emerging evidence for the companion-laden complex participation in ZP binding control, this ubiquitous class of protein may also contribute to activating fusion engines and organizing fusion complexes into functional groups. Several sperm proteins required for fusion with oolemma have functional redundancy, similar to sperm–ZP interactions, on the critical role of chaperone expression during sperm–oolemma fusion and capacitation. For example, getting biologically relevant materials for proteome characterization or performing analyses, such as blue native polyacrylamide gel electrophoresis, has proven challenging in oocytes. HSP70 is produced in preovulatory rat oocytes from oolemma chaperone proteins [89] and mature oocytes from bovine [90]. HSP70 must be activated in two-cell mouse embryos to activate zygotic genes. HSP chaperone protein family found in adult oocytes may be required to translate and fold neonatal proteins during zygotic genome activation properly [91]. The anti-apoptotic activity of HSP70 and another chaperone protein, HSPA5, is unclear.

## HSP70 Expression is Important for Bull Fertility

Bull fertility and HSP70 expression of pathologically defective sperm were hampered by the absence of a critical chaperone, HSP70-2. For HSP70-2 to function, sperm surface receptors capable of interacting with the cumulus–oocyte complex must be assembled and expressed in addition to its other activities [41]. This review highlights the notion that HSP70-2 is involved in male reproduction, coordinating spermatogenesis and the ultimate expression of spermatic function. HSP70-2 was discovered to be required to transition mouse spermatogenic cells from the late-stage to the terminal stage at the testicular level.

Sperm provides the oocyte with more than the paternal genome; it transports the remaining spermatogenesis mRNA [92]. These mRNA transcripts might play roles in early embryonic development [93]. One of the mRNAs with a significant role in fertility is *HSP70*-2. *HSP70*-2 expression level was detected through transcriptomic analysis using quantitative real-time polymerase chain reaction (qRT-PCR). mRNA from semen was used to quantify the HSP70-2 expression level. RNA from sperm was extracted with an RNA extraction kit. DNA contamination could be eliminated using filter-based RNA isolation methods where samples were treated with DNase directly on the filter after lysate binding. The process was followed by cDNA synthesis by converting the extracted RNA sample into cDNA. Subsequently, cDNA was introduced into the qRT-PCR microtitration plate. All models were evaluated in duplicate, and the mRNA expression profile was calculated using the geometric significance of the cycle threshold (Ct). The target genes were normalized using a geometric analysis of two housekeeping genes (*GADPH* and *β-actin*). The difference between the target gene and the reference gene (Ct = Ct of gene target – Ct of reference gene) was used to compute the delta Ct value (DCt) [94].

There are several primers for quantifying HSP70-2 expression using qRT-PCR. Because the structure of the *HSP70-2* gene consists of only one exon (118 bp), it is possible to optimize qRT-PCR conditions using sperm DNA and *HSP70*-2 primer before the expression gene test is carried out. Optimization is carried out in PCR to get the best annealing temperature. Thus, this optimization will make the qRT-PCR process more efficient. Optimization was carried out on the sperm of adult bulls, including Madura cattle. The Madura bull is one of several suspected indigenous Indonesian cattle that hybridize with Zebu cattle (*Bos* *indicus*) and banteng bulls (*Bos* *javanicus*) [95]. Madura cattle are now a breed of locally produced beef cattle. Because of their natural isolation, they exhibit uniform traits that distinguish them from other breeds of Indonesian beef cattle. Zebu cattle have made significant contributions to genetic features, such as climate-induced stress tolerance, tick resistance, and lengthy periods of rigorous natural and environmental selection, contributing to the evolution of additional features.

Initially from dry and barren regions, Madura cattle are an indigenous breed of beef cattle that originated in Madura Island. Weather environment of Madura Island is characterized by heat and dryness and receives only 1600 mm of rain annually. The soil is dry, and the humidity is 80% [96]. These bulls have good heat tolerance, a petite physique, and good reproductive potential. Madura cattle can be used to develop as indigenous beef cattle with an exceptional capacity for adaptation to new situations [97]. HSP70 can also be a good indicator of thermotolerance and thermoresistance [98, 99] and protection against thermal stress in the sperm of Madura bulls. Therefore, HSP70 allows a potential biomarker for an animal’s fertility and protection against heat stress [100]. Thus, *HSP70*-2 is also known as a dual-function gene.

The sperm DNA extraction method was successfully used to detect traces of HSP70-A2 in sperm. Qiagen Blood and Tissue DNA isolation kit (Qiagen, USA) was used to extract DNA from sperm samples according to the manufacturer’s protocol. NanoDrop (NanoDrop Thermo Scientific 200, USA) was used to measure DNA concentration and purity. DNA purity is determined by the A260/280 ratio, which should be 1.8. Purified PCR products were generated using primers reported by Zhang *et al*. [78] in PCR with an annealing temperature of 53°C. The sequences were sorted in First BASE Laboratories (Malaysia). The alignment and comparison of nucleotide sequences were carried out using MEGA 7. BLAST analysis was carried out using the tool on the National Center for Biotechnology Information website (http://www.ncbi.nlm.nih.gov/ncbi.nlm.nih.gov/ncbi.nlm.nih.gov). Multiple sequence alignment was carried out using the Clustal Omega (EBI) website (http://www.ebi.ac.uk/Tools/msa/clustalo/) and the GenBank HSP70-2 accession number NM 174344.1. The results were submitted to EBI for publication.

**Figure-3:**
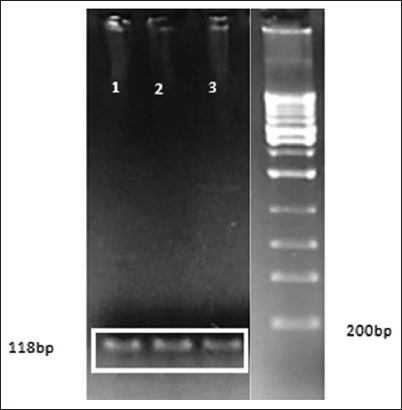
Gel electrophoresis of heat shock protein (*HSP)70-2* polymerase chain reaction (PCR) products. Samples 1 to 3: DNA template (HSP70 gene) from Madura sperm DNA samples. DNA samples were mixed with a 25-μL PCR treatment. Only 3-μL PCR products were conducted on 1× TBE agarose gel in the electrophoresis device (Hoefer) at 60 V for 35 min. Observations were made with UV light with the GelDoc Quantity One program. Source: Rosyada *et al.*, unpublished data.

**Figure-4:**
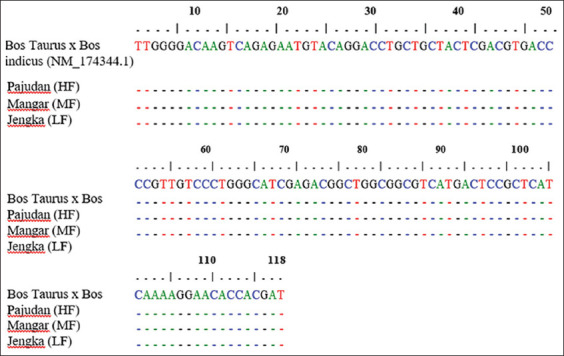
Sequence alignment of the heat shock protein (*HSP)70-2* gene in frozen semen of Madura cattle post-thawing using MEGA 7 version 0.26 [Source: Rosyada *et al.*, unpublished data].

PCR products were detected on 1.2% ethidium bromide (EtBr) agarose gel using 1× TBE buffer (89-mM Tris, 89-mM boric acid, and 2-mM Ethylenediaminetetraacetic acid, pH 8.0) on the electrophoresis device (Hoefer, USA). Observations were carried out with ultraviolet light with the GelDoc Quantity One (Bio-Rad) program after the gel was stained with EtBr (0.5 g/mL). Electrophoresed DNA samples were analyzed by comparing the sample DNA band with the DNA ladder bands and verified with sequencing results. The size of the HSP70-2 PCR product was 118 bp (Figure-3). The forward and reverse sequencing results of the Madura bull *HSP70-2* gene were aligned with *HSP70-2* of the *Bos taurus* bull gene in GenBank (accession no. NM_174344.1; Figure-4).

**Figure-5:**
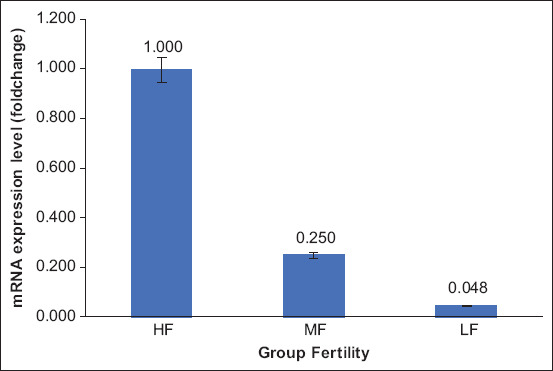
Heat shock protein 70-2 mRNA expression levels in bovine sperm collected from Madura bulls with high fertile, medium fertile, and low fertile quality sperm. Significant differences (p < 0.05) [Source: Rosyada *et al.*, unpublished data].

HSP70-2 gene sequence alignment results in Madura bulls using MEGA 7 version 0.26 and BioEdit are shown in Figure-5. Based on the sequencing results, the *HSP70-2* gene was identified in post-thawing frozen semen of Madura bulls. The sequence alignment of *HSP70-2* identification on sperm is also supported by the isolation of Madura sperm RNA, examining variations in sperm mRNA among high, medium, and poor fertile semen-producing Madura bulls to see if *HSP70-2* transcript could be a valid diagnostic tool of semen quality. These data are an example of the application of *HSP70-2* effects on Madura bull semen. Data in this review strengthen the data in other similar studies. The relative mRNA expression level of HSP70-2 was higher in the high fertile Madura bull group than in the low fertile Madura bull group (Figure-5).

## Conclusion

Based on the reviews from several journals and articles regarding the role of HSP70-2 on bull fertility, it is well established that HSP70 belongs to a large family of chaperone proteins with important functions. *HSP70-2* is part of a larger network that includes other HSP family members. HSP protects cells from the effects of internal and external stressors. HSP plays several vital roles, the molecular and cellular details of which in reproduction and fertility are only now being addressed. Impaired HSP70 expression in spermatozoon interferes with fertilization. Still, there is a lack of knowledge in the molecular underpinnings and mechanisms and thereby a need for further research on HSP70 as a sperm quality molecular biomarker.

Research suggested that HSP70-2 may accommodate sperm thermotolerance to acute or chronic changes in temperature (environmental or associated with sperm processing). Madura cattle are produced in Madura Island, Indonesia, with dry and tough conditions. Madura bulls can adapt to thermal stress and poor diet and have a small body with high fertility. Further studies on Madura bull sperm and *HSP70-2* expression in cells can lead to discovering potential markers and mechanisms of sperm thermotolerance or thermoresistance. Findings related to HSP and HSP70-2 can help uncover fertility biomarkers that can be used as diagnostic tools in cattle and other mammals and select bulls with environmental and heat adaptability.

## Authors’ Contributions

ZNAR: Conceived the idea and drafted and revised the manuscript. MFU and LITAT: Reviewed the manuscript. DDS, BP, and EM: Literature search and edited and reviewed the manuscript. All authors read and approved the final manuscript.
